# Force measurement goes to femto-Newton sensitivity of single microscopic particle

**DOI:** 10.1038/s41377-021-00684-6

**Published:** 2021-12-08

**Authors:** Xiaohe Zhang, Bing Gu, Cheng-Wei Qiu

**Affiliations:** 1 Advanced Photonics Center, Southeast University, Nanjing, 210096 China; 2 Department of Electrical and Computer Engineering, National University of Singapore, 4 Engineering Drive 3, Singapore, 117583 Singapore

**Keywords:** Optics and photonics, Applied optics

## Abstract

Highly sensitive force measurements of a single microscopic particle with femto-Newton sensitivity have remained elusive owing to the existence of fundamental thermal noise. Now, researchers have proposed an optically controlled hydrodynamic manipulation method, which can measure the weak force of a single microscopic particle with femto-Newton sensitivity.

Micromanipulation techniques have experienced impressive progress for manipulating and trapping microscopic objectives. Common micromanipulation techniques include optical, magnetic, electrokinetic, acoustic, thermophoretic, and hydrodynamic tweezers^1–3^. Different technologies have their own advantages and disadvantages. The optical and magnetic tweezers have high requirements on material properties^4^. In contrast, the electrokinetic and hydrodynamic tweezers can manipulate the particle without restrictions on the material properties. However, most of these techniques have a relatively low spatial resolution. Optical tweezers may not be suitable for long-time trapping of objects owing to the photo-induced damage or heating^4^. Compound manipulation is the most effective way to alleviate these limitations, such as magneto-optical, opto-thermophoretic, and opto-hydrodynamic^5–7^. Based on the optical and hydrodynamic manipulations, the optically controlled hydrodynamic manipulations have been proposed to trap and observe microscopy objects^6,7^. This method does not expose the object to the high-intensity optical field, while also lifting material constraints.

How to improve the sensitivity of measurement techniques is a major concern for many experimental scientists. In the past few decades, a variety of methods for weak force measurements have been proposed, including micro-electro-mechanical force sensors, fiber-optic force sensors, nanocantilevers, and atomic force microscopy^8–10^. These weak force measurement methods exhibited an unambiguous range of ~0.6 mN and an ultra-small detection limit down to ~0.1 pN^2^. These force measurement methods face a common problem: the fundamental thermal noise related to the motional degree of freedom^11^. Therefore, it is difficult to achieve the detection of the weak force of a single microscopic particle at femto-Newton level.

A recent research paper in *eLight*^12^, entitled “Highly sensitive force measurements in an optically generated, harmonic hydrodynamic trap”, by Iliya D. Stoev from Max Planck Institute of Molecular Cell Biology and Genetics, provides an optically controlled trapping method enabled by light-induced hydrodynamic thermoviscous flows in a thin microchannel. By splitting the scanning line of an infrared laser into two counter-directed paths on the same axis, they transition from a flow dipole to a quadrupole-like flow-field making use of opposite collinear thermoviscous flow fields. Specifically, a stagnation point is formed between the two counter-directed laser scan paths in a region without direct laser exposure (Fig. 1b). By using a feedback control system (Fig. 1a), they effectively create a quasi-1D-trapping situation. As shown in Fig. 1c, these four particles are always to be displaced along the compressional axis only. Furthermore, they investigate the relationship between the velocity and distance of a 3 μm trapped polystyrene particle. Interestingly, they observe an exponential approach to the stagnation point, suggesting a linear force-displacement relationship (Fig. 1d). They also measure the properties of the trap, showing a femto-Newton range force measurement with sensitivity close to the thermal limit. In addition, this optofluidic trap is highly tunable, including the laser power, the scan path length, or the counterflow. Their force measurements eliminate the material constraints and also remove the need for laser-particle contact. This optofluidic trap is an appealing alternative or complement to optical tweezers for probing the active microrheology of weak viscoelastic networks. These results obtained by this manuscript open the exciting possibilities of ultrasensitive force measurements of individual nanoparticles. The optically controlled hydrodynamic manipulation with ultrasensitive will certainly be widely used in the life sciences and engineering.

**Fig. 1:**
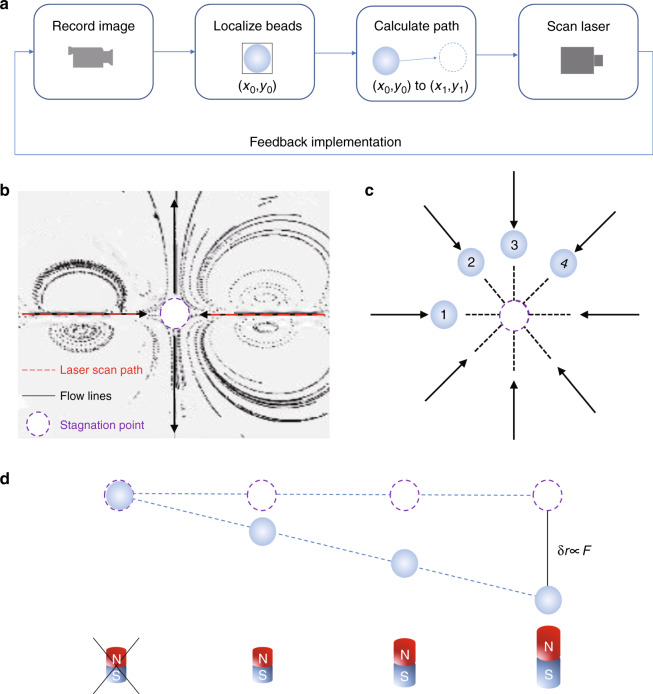
Concept and implementation: Utilizing the scanning laser to create a hydrodynamic trap to measure the force. **a** System schematic diagram of closed feedback loop operation to automatically position particles. **b** Visualization of generated flow-field lines, laser scan path, and stagnation point. **c** The resorting character of the thermoviscous flows (black arrows). **d** Linear relationship between the force and displacement. (Fig. 1a and 1b adapted and re-plotted based on Fig. 1c in ref. ^13^ and Fig. 1b in ref. ^12^, respectively.)

Although optical micro/nanomanipulations are advancing rapidly in the past few decades, there are still some grand challenges that deserve more attention and effort to address. First, we still need to identify effective approaches to realize strong manipulation under moderate light or even without direct contact with light exposure. Different degrees of freedom and mechanisms, other than just intensity gradient, are to be studied. Second, extraordinary multipoles of high-index dielectric particles provide unprecedented optical force and torques. This should be an interesting topic in biological and quantum sensing. Third, it is still largely elusive how to achieve multifunctional manipulation of arbitrary single or massive tiny bioparticles (e.g., viruses) in a solution.
